# Virchow on Cholera

**Published:** 1885-07

**Authors:** 


### Virchow on Cholera.

A series of popular articles in *Die Nation*, a weekly political journal of Berlin, condemns the French for carelessness, and takes a conservative view of the significance of the cholera bacillus. In a speech at the public reception of the Cholera Commission on their return to Berlin, he said: "It appears to me that the government is not entirely free from the opinion that with the discovery of the bacillus everything is accomplished which may be necessary to control the disease. * * * The tubercle bacillus is a very important thing, but, with the exception of a new point of view of the disease, we are no further advanced in our practical relations to it." He said that cholera had long been treated as if caused by a special organism. He referred also to the laxity of English quarantine.

